# Fabrication and Characterization of PCL/HA Filament as a 3D Printing Material Using Thermal Extrusion Technology for Bone Tissue Engineering

**DOI:** 10.3390/polym14040669

**Published:** 2022-02-11

**Authors:** Fengze Wang, Esma Bahar Tankus, Francesco Santarella, Nadja Rohr, Neha Sharma, Sabrina Märtin, Mirja Michalscheck, Michaela Maintz, Shuaishuai Cao, Florian M. Thieringer

**Affiliations:** 1 MIRACLE Smart Implants Group, Department of Biomedical Engineering, University of Basel, 4123 Allschwil, Switzerland; fengze.wang@unibas.ch (F.W.); esma.tankus@unibas.ch (E.B.T.); francesco.santarella@unibas.ch (F.S.); neha.sharma@usb.ch (N.S.); mirja.michalscheck@unibas.ch (M.M.); michaela.maintz@unibas.ch (M.M.); 2 Medical Additive Manufacturing Research Group (Swiss MAM), Department of Biomedical Engineering, University of Basel, 4123 Allschwil, Switzerland; 3 Biomaterials and Technology, Department of Reconstructive Dentistry, University Center for Dental Medicine Basel UZB, University of Basel, 4058 Basel, Switzerland; nadja.rohr@unibas.ch; 4 Clinic of Oral and Cranio-Maxillofacial Surgery, University Hospital Basel, 4031 Basel, Switzerland; 5 Biomaterials and Technology, Department of Research, University Center of Dental Medicine Basel UZB, University of Basel, 4058 Basel, Switzerland; sabrina.maertin@uzb.ch; 6 Institute for Medical Engineering and Medical Informatics, University of Applied Sciences and Arts of Northwestern Switzerland, 4132 Muttenz, Switzerland; 7 Department of Stomatology, Shenzhen University General Hospital and Shenzhen University Clinical Medical Academy, Shenzhen University, Shenzhen 518071, China

**Keywords:** polycaprolactone (PCL), hydroxyapatite (HA), material extrusion, three-dimensional printing, scaffold, hydrophilicity, mechanical testing

## Abstract

The most common three-dimensional (3D) printing method is material extrusion, where a pre-made filament is deposited layer-by-layer. In recent years, low-cost polycaprolactone (PCL) material has increasingly been used in 3D printing, exhibiting a sufficiently high quality for consideration in cranio-maxillofacial reconstructions. To increase osteoconductivity, prefabricated filaments for bone repair based on PCL can be supplemented with hydroxyapatite (HA). However, few reports on PCL/HA composite filaments for material extrusion applications have been documented. In this study, solvent-free fabrication for PCL/HA composite filaments (HA 0%, 5%, 10%, 15%, 20%, and 25% weight/weight PCL) was addressed, and parameters for scaffold fabrication in a desktop 3D printer were confirmed. Filaments and scaffold fabrication temperatures rose with increased HA content. The pore size and porosity of the six groups’ scaffolds were similar to each other, and all had highly interconnected structures. Six groups’ scaffolds were evaluated by measuring the compressive strength, elastic modulus, water contact angle, and morphology. A higher amount of HA increased surface roughness and hydrophilicity compared to PCL scaffolds. The increase in HA content improved the compressive strength and elastic modulus. The obtained data provide the basis for the biological evaluation and future clinical applications of PCL/HA material.

## 1. Introduction

Oral and maxillofacial tumors and trauma often lead to different degrees of jaw defects, and their reconstruction remains a challenging task [1]. Autogenous or allogeneic bone transplants are commonly used to rehabilitate jaw defects [2,3,4]. Due to the limited availability of bone material, various biomaterials have been applied to generate scaffolds using three-dimensional (3D) printing [5]. Among the biocompatible materials, polycaprolactone (PCL) has attracted much in bone tissue engineering and is widely used in 3D printing, enabling the fabrication of complex patient-specific and biomimetic structures [6]. PCL is a resorbable polymer with a low production cost, and it can be combined with osteoconductive materials such as hydroxyapatite (HA) [7,8,9]. Composites comprise two or more materials, and the goal is to create more efficient scaffolds by combining the regenerative properties of more than one biomaterial [10]. Furthermore, composites containing HA and polymers combine good mechanical properties with good biocompatibility, yielding a 3D substitute that mimics the heterogeneity and hierarchical structure of the native extracellular bone matrix [11,12].

Fabrication methods for PCL/HA porous biodegradable polymer scaffolds include thermally induced phase separation (TIPS), salt leaching, gas forming, freeze-drying, and 3D printing technology [13,14]. However, compared with the above methods, only 3D printing technology enables the control of the internal structures of complex implants [15]. This technique is currently being explored for surgical bone replacement [16]. Three types of 3D printers have been considered for bone reconstruction: stereolithography (SLA), material extrusion, and selective laser sintering (SLS). SLA uses only photoactivatable biopolymers [17], whereas the SLS method suffers from a limited choice of materials [18]. Compared with other 3D printing technologies, material-extrusion is a scalable industrial route that does not require a solvent and optimizes the filler (HA) distribution [19].

With material extrusion printing, a prefabricated filament is loaded and extruded from a hot nozzle to a workbench. The extruded filament hardens at room temperature with cooling fans to form a solid structure in a layer-by-layer pattern [20]. Material extrusion printing is suitable for printing patient-specific implants and easy-to-scale manufacturing technology, and constitutes the vast majority of the 3D printer market. However, the material extrusion method has not been extensively investigated for scaffold fabrication in maxillofacial reconstruction [21,22]. Melting material for the fabrication of a single composite filament is an affordable and cost-effective method. The procurement cost of PCL powder is about USD 2 per gram, whereas HA powder is USD 4 per gram [23]. Hence, PCL/HA filament fabrication material costs were around USD 2.1 to 2.5 per gram, depending on the proportion (0–25%) of HA added. Filament fabrication favorably uses a thermal procedure, having the advantage of producing solvent-free and eco-friendly filaments with no harmful effects (such as cell death) derived from solvent residuals [24,25]. Although solvent-casting techniques are commonly used to fabricate PCL composite filaments, these techniques suffer from inherent limitations, such as the challenging fabrication process associated with the leaching of solvent residues in the fabricated scaffolds; thus, it might not be suitable for scaffold fabrication in hospital settings [26,27,28,29]. A hot-melt filament extrusion machine would include HA and fabricate the composite filament in a cost-contained situation [30,31]. Nonetheless, after obtaining a successful filament, the optimum build orientation, layer thickness, nozzle diameter, infill pattern, and bed temperature are necessary to fulfill this need [32]. Hot-melt PCL/HA filament fabrication and the scaffolds fabricated from this process are understudied; to date, only scant information is available on the fabrication settings for machines in research environments [33]. Additionally, further studies should be conducted to assess the optimal HA percentage for osteoconductive applications.

This study aimed to test the feasibility of filament fabrication for an osteoconductive material (PCL-HA) and printing parameters with an affordable material on an extrusion-based 3D printer. Filaments of PCL with different HA percentage contents were used to test the effect on printing parameters. Compressive strength, elastic modulus, water contact angle, and morphological analysis parameters were obtained as quality indications for implantability.

## 2. Materials and Methods

### 2.1. PCL and HA Composite Filament Fabrication

**Figure 1:**
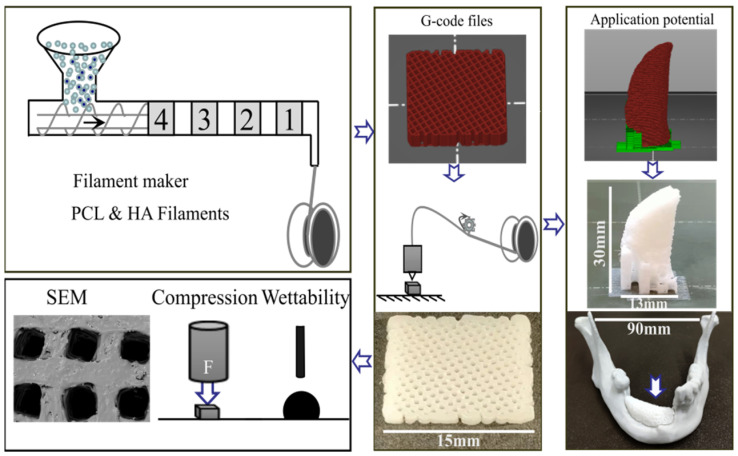
Filament fabrication and material extrusion-based 3D printing. 4, 3, 2, 1: separate zones in the filament-maker. The new composite filament is collected and used for 3D printing following the represented G-code file, giving a product of 15 × 15 × 2 mm^3^. The printed scaffold is tested for SEM (scanning electron microscopy), compression, and wettability properties. The same filament is used for potential application in the bone replacement model.

Six different weight proportions of HA (average powder size: 15 μm, Nanjing Emperor Nano Material Co., Ltd., Nanjing, China) 0%, 5%, 10%, 15%, 20% and, 25% *w/w* were mixed with PCL (molecular weight: 50,000 g/mol, powder size: 120 μm, ρ = 1.146 g/mL, Gao Ju material Co., Ltd., Guangzhou, China). PCL and HA materials were dried in an AIRID Polymer dryer (3devo, Utrecht, Netherlands) for 3 h at 40 °C. The dried material was placed in the hopper of the filament-maker (3devo, Utrecht, Netherlands). The starting values were initially set as detailed in the PCL manufacturers’ instructions and based on previous publications [34,35]. Four temperature gradients (T4–T1, where T4 is the first melting spot closest to the hopper, followed by T3, T2, then T1, the exit melting point), were adjusted to 10% higher than the PCL’s declared melting temperature (60 °C → 66 °C) and the extruder rotational speed of the single-screw extruder was initially adjusted at 5 RPM to ensure that material can be extruded. Filaments that did not comply with a circular shape were excluded. Filament cooling was performed with a dual-fan system (cooling fan speed at 100%) to achieve solidification. The final four melting zone temperatures (T4–T1) of each group were determined by decreasing the temperature by 1 °C in each cycle and decreasing the extrusion speed (RPM) until the diameter stabilized at 1.75 ± 0.05 mm, and were considered a functional diameter for material extrusion 3D printing [34]. The filament was then linked to a puller to collect the product (Figure 1). After the puller had stabilized the diameter of the filaments within the target diameter range for 10 min, the winder was set to spool the filaments. Filaments were then stored in Ziploc bags and dried for 15 min with a vacuum dryer (Piston pump 406G, Reciprotor, Denmark) before printing. Extrusion speed refers to the screw rotational speed (RPM) controlled by the 3devo machine, whereas the filament extrusion speed was calculated on the extruded PCL filament length per second (mm/s).

### 2.2. 3D Printing of Scaffolds

A 3D scaffold model was developed (Tinkercad, Autodesk, San Francisco, CA, USA, and PrusaSlicer, Version 2.3.1, Prague, Czech Republic). The parameters of the G-code file included the layer height (100 μm) with an infill density of 90%. The infill pattern was chosen as a grid with an infill angle named in the software as “0/120°” to avoid interfilamentous distance closure due to melting. Subsequently, the six different groups’ scaffolds were printed using a printer (Prusa Mini, Prague, Czech Republic) with a nozzle of 400 μm (Figure 1). After loading the filament into the Prusa Mini printer, parameters, such as the printing speed, nozzle temperature, heat bed temperature, and nozzle flow factors (amount of material flowing through the nozzle) were adjusted, and the print fan speed was set to 255 RPM at room temperature (20 °C) to cool the scaffolds. Three different sizes of six groups’ scaffolds were printed—square solid specimens (15 × 15 × 2 mm^3^, *n* = 5, for surface roughness measurements and water contact angle test), square porous scaffolds (15 × 15 × 2 mm^3^, *n* = 3, for morphological analysis) and square porous scaffolds (15 × 15 × 4 mm^3^, *n* = 3, for the mechanical test)—according to the test needed. After production, scaffolds were stored in a desiccator with silica gel before use.

### 2.3. Surface Roughness and Pore Size Quantification

Five solid specimens for each group were printed to measure the surface roughness parameters using a 3D laser scanning microscope (VK-X-1050, magnification 50×, Keyence, Osaka, Japan). Three sites of 200 μm × 200 μm per scaffold were obtained, and after surface shape correction and height cuts, a 0.8 μm Gaussian S-filter was applied. The arithmetical mean height (Sa) and the maximum height of surface (Sz) were extracted using the multi-file analyser software (Version 2.1.3.89). The pore size of porous scaffolds (*n* = 3) was determined by considering 30 pores for each scaffold using a 3D laser scanning microscope [9]. The porosity of the scaffolds was obtained following the equation: porosity (%) = 1-scaffold density/arithmetic mean density. The scaffold density was defined as the weight of the scaffold divided by the volume of the scaffold. The mean densities of PCL and HA were 1.146 g/cm^3^ and 3.16 g/cm^3^, respectively [36].

### 2.4. Scanning Electron Microscopy (SEM) and Energy-Dispersive X-ray Spectroscopy

Images of the surface morphology of the porous scaffolds were obtained using scanning electron microscopy (SEM, XL30, Philips, Eindhoven, The Netherlands) at an accelerating voltage of 10 kV and 50× magnification. Additionally, energy-dispersive X-ray spectroscopy (EDX) was performed to determine the elements present on the surface of the scaffolds at 20 kV.

### 2.5. Water Contact Angle

The hydrophilicity of the scaffold materials was tested on three solid specimens per group using a drop shape analyzer (DSA100, Krüss, Hamburg, Germany). The specimens were cleaned in an ultrasonic bath (TPC-15, Telsonic Ultrasonic, Bronschhofen, Switzerland) with 70% ethanol for 4 min. Three 2 μL drops of ultrapure water were applied to each specimen using the sessile drop technique. After drop placement, the angle between the specimen surface and the contour of the drop was analyzed as the contact angle. The mean of the right and left contact angles were calculated for each specimen.

### 2.6. Mechanical Test

The compressive strength of the scaffolds (15 × 15 × 4 mm^3^) was evaluated using a universal testing machine (Z020, Zwick/Roell, Ulm, Germany) at a crosshead speed of 1 mm/min. Compressive strength (MPa) was calculated as the maximum applied load (N)/compressed area of the surface (mm^2^) after a 20% deformation of the scaffolds. The elastic modulus of the samples was additionally recorded between 2% and 5% deformation.

### 2.7. Statistical Analysis

Data were expressed as the mean ± standard error of the mean (s.e.m). The data were analyzed with a one-way analysis of variance (ANOVA), and post hoc Tukey’s test was performed to compare the statistical difference. The level of significance was set to *p* < 0.05. All statistical analyses were performed using GraphPad Prism 8.0 (GraphPad, San Diego, CA, USA).

## 3. Results

### 3.1. Filament Fabrication

Filament extrusion was successfully achieved by tuning the four melting points of the filament-maker (3devo) (Table 1). The PCL melting temperature was gradually increased by increasing the amount of HA. The first melting point (T4) allowed a pre-melting of the PCL powder; therefore, this PCL temperature plus 10% variation was set when supplemented with HA powder. The intermediate melting points (T3 and T2) were set at higher temperatures, from 64 °C to 70 °C, to allow interspersion of HA particles into the PCL matrix. The exit melting point temperature (T1) was intended to achieve correct filament extrusion from the filament-maker nozzle. T1 was generally at a lower temperature than the other points. The extruder rotational speed was initially set at 5 RPM and reduced to a range of 2—2.9 RPM on a trial-and-error basis. The applied parameters for each 5% increase in HA content in PCL are displayed in Table 1. In general, 61 °C to 69 °C was required when HA was present in the powder mix versus 60 °C when pristine PCL powder was used.

### 3.2. Printing Procedure

**Figure 2:**
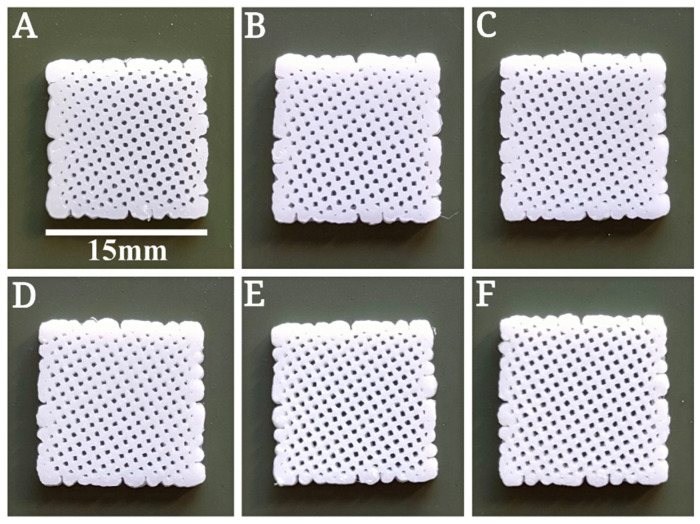
Examples of printed six groups’ scaffolds (15 × 15 × 2 mm). (**A**) PCL. (**B**) PCL + 5% HA. (**C**) PCL + 10% HA. (**D**) PCL + 15% HA. (**E**) PCL + 20% HA. (**F**) PCL + 25% HA.

Table 2 describes which temperature, speed, and flow factors can be used according to the HA content. From pure PCL filament to 25% HA content, an increase of 30 °C, and minor fluctuations in printing speed and flow factor were recorded. The applied parameters reported in Table 2 were used to print the six groups’ scaffolds, maintaining a mean pore size of 550 µm (Section 3.3). These parameters describe the temperature required to melt the filament, ranging from 174 °C to 205 °C. We fabricated ten scaffolds per group to verify the above parameters, and the results showed that the parameters were stable and reliable with a 100% success rate (Figure 2 and Supplementary Figure S3).

### 3.3. Specimen Morphological Characterization

**Figure 3:**
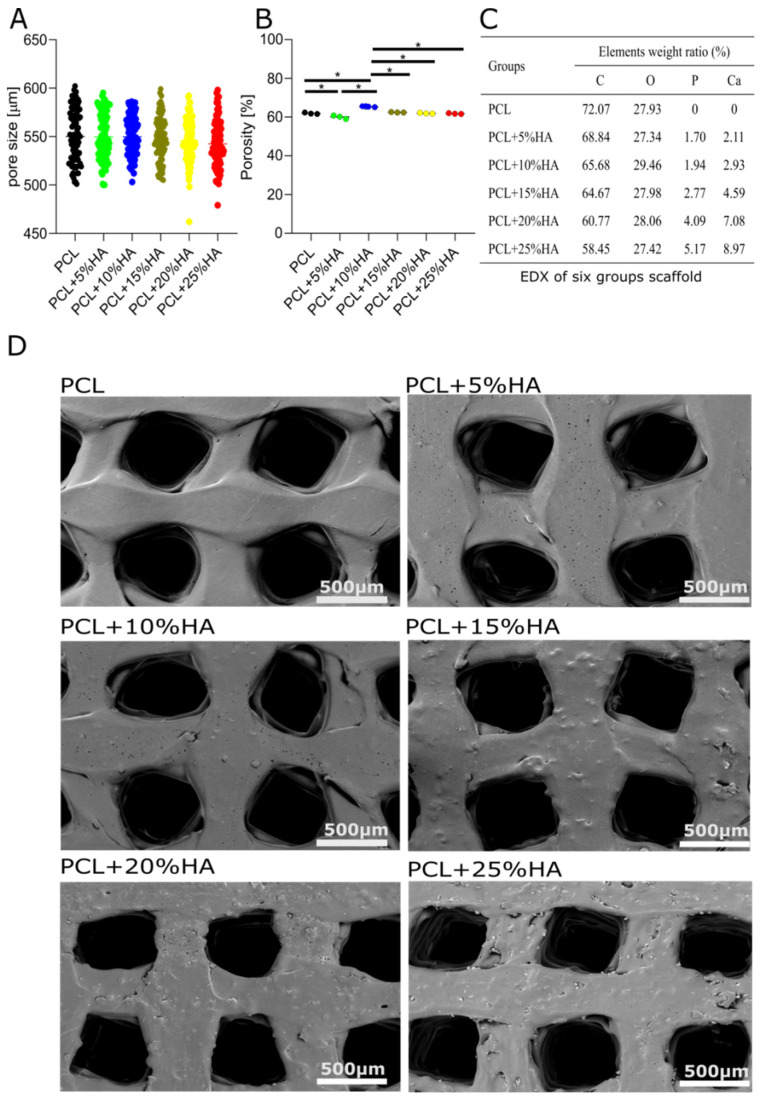
Surface characterization of the scaffolds. (**A**) The pore size of the six groups of scaffolds (mean ± s.e.m); 90 measurements of 3 scaffolds in each group (one-way ANOVA + Tukey’s post hoc test, n.s. = *p* > 0.05). (**B**) Porosity was calculated based on the weight of the scaffolds. The graph represents the mean ± s.e.m (one-way ANOVA + Tukey’s post hoc test, * = *p* < 0.05, *n* = 3). (**C**) Energy-dispersive X-ray spectroscopy (EDX) of the different groups (complete scaffold surface scanning). (**D**) Scanning electron microscopy (SEM) image of the surface of the scaffolds. HA granules are visible in the structure: scale bar = 500 μm.

The pore sizes of scaffolds containing different HA content remained constant (not significantly different from 550 µm). The porosity rate exhibited a smaller oscillation range, with PCL + 10% HA showing the highest porosity (65.4 ± 0.3%, * = *p* < 0.05) and PCL + 5% HA the lowest (60% ± 0.9%, * = *p* < 0.05) versus the control and the other groups (Figure 3A,B). Despite the statistical significance, the porosity of all six groups’ scaffolds was around 60%. SEM was used to obtain the morphology (Figure 3D). The images revealed that all six groups of scaffolds displayed highly interconnected pore structures. Additionally, the structure appeared rougher the more HA was included. To verify whether this correlated with the increased incorporation of HA, EDX was conducted for each group. From the EDX, we analyzed the percentage of HA on the surface of scaffolds. The EDX analysis revealed that the weight ratio of calcium (Ca) and phosphorus (P) increased concomitantly with the increasing amounts of HA included (Figure 3C).

### 3.4. Specimen Roughness Characterization

**Figure 4:**
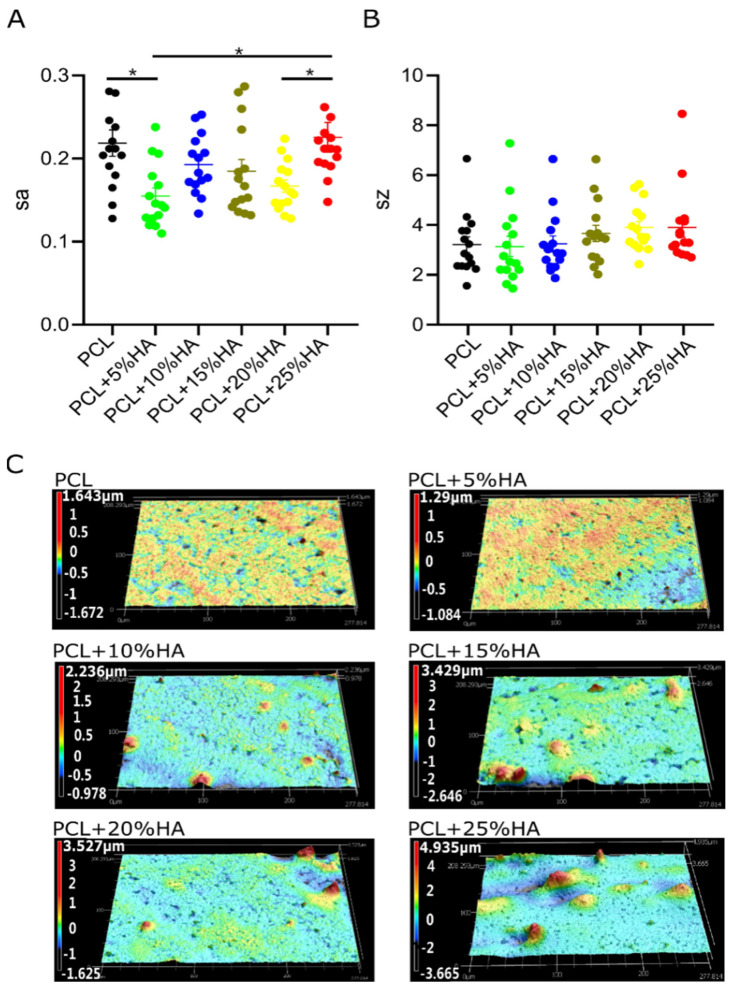
Surface roughness analysis of the six groups’ scaffolds. (**A**) Graph on the left side represents Sa (arithmetical mean height); (**B**) Graph on the right represents Sz values (maximum height of surface). Graphs show mean ± s.e.m, * = *p* < 0.05, one-way ANOVA + Tukey’s post hoc test (*n* = 5). (**C**) Surface topography of the six groups. Pseudo-color blue = valleys, pseudo-color red = peaks.

Surface roughness parameters Sa (arithmetical mean height) and Sz (maximum height) are displayed in Figure 4. The PCL + 25% HA group showed the highest mean Sa (ca.0.25), and the PCL + 5% HA group showed the lowest mean Sa value (ca.0.15), with statistical differences from the control PCL (* = *p* < 0.05). Regarding Sz, although no statistical differences were recorded between the groups (n.s. = *p* > 0.05), the values increased with the increased HA content.

### 3.5. Water Contact Angle Test

**Figure 5:**
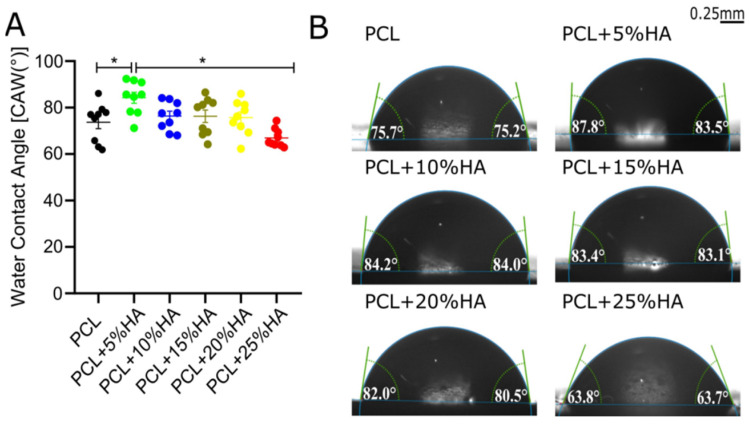
Water contact angle test. (**A**) Graph represents the water contact angle, mean ± s.e.m, * = *p* < 0.05 (one-way ANOVA + Tukey’s post hoc test (*n* = 9). (**B**) Images of water contact measurements for each group left and right side: scale bar = 0.25 mm.

Water contact angles were highest for the PCL + 5% HA group (84 ± 7.2°, * = *p* < 0.05) versus the PCL control. In contrast, the PCL + 25% HA group showed the best hydrophilic performance (67 ± 3.9°, * = *p* < 0.05) versus the PCL + 5% HA group (Figure 5A). Contact angles of intermediate HA content were not statistically significant from the PCL control. Water drops on all solid specimens are displayed in Figure 5.

### 3.6. Mechanical Properties

**Figure 6:**
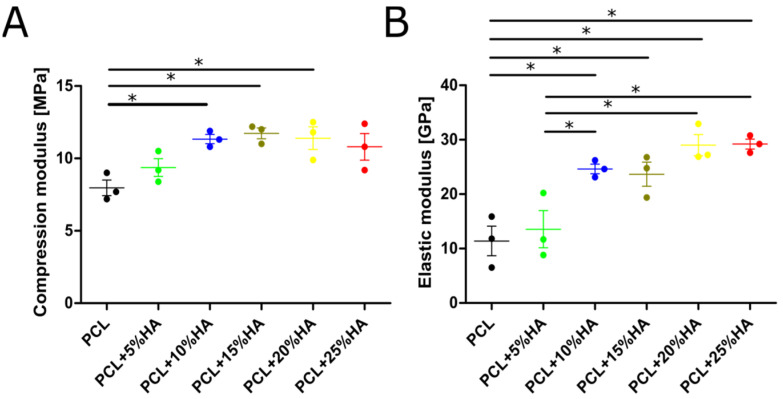
Mechanical test of the scaffolds. (**A**) Compressive strength after 20% deformation (MPa). (**B**) Elastic modulus obtained between 2% to 5% deformation (GPa). Graphs represent mean ± s.e.m, one-way ANOVA paired data + Tukey’s post hoc test, * = *p* < 0.05.

Every scaffold was tested until it reached 20% deformation. The PCL control group showed the lowest MPa value (8 MPa). Compression values proportionally increased with 5% and 10% HA contents (to 9 and 11 MPa, respectively). When comparing PCL + 10%, 15%, and 20% HA groups, they were statistically significant compared with the control (*p* < 0.05), but not compared with each other, reaching a plateau of compression around 11 MPa (Figure 6A). The 25% HA group only exhibited significance approaching that of PCL. The elastic modulus increased with increasing HA content, following the same trend seen in the uniaxial compression tests. Scaffolds including 10%, 15%, 20%, and 25% HA had a mean elastic response between 24 and 30 GPa (Figure 6B).

## 4. Discussion

Bone defects due to cancer, trauma, or fracture remain a considerable clinical challenge and require bone grafting. It has been estimated that over two million bone implant surgeries are conducted worldwide each year [37]. Many problems may arise from these bone implant surgeries, including infection, host rejection, model fitting, and material scarcity; thus, alternative methods and new materials are increasingly being developed [38]. Three-dimensional printing is a suitable alternative to producing personalized grafts [39]. Having a material extrusion system available for graft printing can drastically reduce the costs and time required for implant surgery across the globe, improving the accessibility to grafts for more countries [40]. Surprisingly, the material extrusion method has rarely been used for scaffold fabrication, probably due to the lack of suitable materials and control methods [21]. Historically, solvent casting has been the main method, with only a few studies focusing on hot-melt extrusion, although using higher HA contents which, in return, influences printability parameters, involves the use of more expensive printers, and increases the presence of cell byproducts [26,27,28,29,33].

In the first step, parameters to fabricate PCL/HA filaments were fixed in this study. Due to its good biocompatibility, slow degradation, and FDA approval, PCL has become the most commonly used thermoplastic polymer [41,42,43]. In addition, PCL degradation products are not toxic and can be eliminated by the human metabolic system [10]. However, its hydrophobic and non-osteoinductive properties limit PCL’s applications as clinical implant material [44]. Therefore, PCL is usually applied in combination with calcium phosphate ceramics, such as HA, displaying appropriate mechanical properties with hydrophilicity and biocompatibility for a bone scaffold [45]. It was found that each percentage of HA included in the filament-maker required at least a 1 °C difference in melting, for up to 25% HA. This can be related to the increase in material viscosity caused by HA. As the viscosity increases, the risk of printer nozzle clogging increases too. Therefore, the highest permissive HA concentration was set to 25%. Most studies used a ceramic content of less than 30% to avoid brittleness and the degradation of mechanical properties caused by great concentrations [46]. This requires validation when changing components (i.e., bone inducer β-TCP instead of HA) [47]. In addition, the extrusion speed was vital to filament fabrication. High speed (5 RPM) resulted in rapid extrusion and thick filament segments (See Supplementary Figure S1 and Supplementary Table S1). Limiting the speed to a maximum of 2.9 RPM yielded the desired filament thickness (1.75 ± 0.05 mm diameter, used by most material-extrusion-based 3D printers) and no thicker HA-clustered segments in our test.

In addition to filament fabrication, the literature suggests ensuring full control over the filament deposition temperature and interfilamentous distance is required [32,48]. In this paper, speed, and temperature were the key parameters monitored. The percentage of HA influenced the filament melting deposition temperature. It was found that there was a 5 °C difference among formulations to maintain our scaffolds’ pore sizes constant, at about 550 µm (calculated from two optimal bone size ranges: 150 μm to 600 μm [49,50], and 450 to 700 μm [51]). Pore sizes larger than 300 μm would effectively promote vascularization, cell growth, and infiltration within the scaffold [13,52,53]. Viscosity was affected by the HA content; therefore, the speed and temperature were adjusted to prevent pore closure. This temperature was three times higher than the filament fabrication temperature. The reason was that the nozzle in the desktop 3D printer had an extrusion diameter of 0.4 mm. The nozzle melted the hard filament and left the powder in a shorter time and distance as compared to the four melting zones. The design parameter “infill angle” was essential to prevent pore occlusion. Different deposition patterns would produce other PCL scaffold pore structures [54]. The finding showed that pores were occluded if 0/45° or 0/90° infill angles were used, which was prevented by using 0/120°. From Figure 3D, it can be observed that HA is homogeneously distributed in PCL. The final pore size and porosity of all 3D-printed scaffolds were designed through a software approach. Polymers such as PCL/HA (not metallic or inorganic materials) will melt to form specific structures during the printing process due to the material’s properties, exhibiting higher viscosity than the PCL control sample, and consequently, a higher printing fidelity. Every other parameter (temperature, speed, and flow factor) which did not generate a suitable scaffold (different from the 550 µm mean pore size), was automatically excluded (Supplementary Figure S2 and Supplementary Table S2). Excluded scaffolds and parameters were tested and those which failed were either not extruded (temperature too low), too much extruded (temperature too high, low speed, or high flow factor), or the pores were interrupted (high speed or low temperature) (Supplementary Figure S2). Until now, the precise parameters for PCL/HA concentration had only partially been reported and limited to successful scaffold fabrication to expensive 3D printers [33]. We have precisely described the temperatures and speed required for each composition, reporting the causes of failure in the fabricated scaffolds. These parameters are of great importance for future clinical references, by using entry-level equipment.

To yield a homogeneous product, HA should be evenly distributed to ensure progressive smoothness and wettability with increasing content in the printing. Surface roughness analysis, Sz and Sa, revealed homogeneity, starting from 10% HA content, similar to what was found in the PCL + 10% β-TCP group and in previous implants [55,56]. In support of this, the smallest contact angle, reflecting better hydrophilicity, was also found at higher HA concentrations (ca. 78° at 20% HA), in accordance with previous findings [57]. The major weakness of PCL was its hydrophobicity, which is not favored by osteoblasts and endothelial cells, and thus is not conducive to the formation of an osteogenic and vascular microenvironment. However, by adding HA, the surface roughness and hydrophilicity of the material were changed, which may affect cell adhesion and proliferation. Ceramic particles tend to exhibit better hydrophilicity than polymer components due to their inorganic components [58]. As an exception, we found that the PCL + 5% HA sample did not follow this rule (Figure 4). PCL + 5% HA outperformed PCL in the hydrophobicity assays, in correlation with its lower surface roughness [59].

Regarding the mechanical properties, the higher the modulus of elasticity, the less likely a graft will undergo deformation. PCL scaffolds could withstand the least force and were more prone to deformation. The PCL + 20% scaffold could resist pressure and was the least prone to deformation. Additionally, the E-modulus (11.4–29.2 GPa) and compressive strength (8–11.7 MPa) for the six groups of samples were at the lower limit of mandibular trabeculae values (6.9 to 199.5 MPa and from 0.22 to 10.44 MPa, respectively) [60,61]. Taking all the results into consideration, future projects will focus on the biological analyses of candidate scaffolds with different HA contents. The elastic modulus results indicate a linear increase with increasing amounts of HA, stagnating at PCL + 10% HA. Further experiments with increasing amounts of HA need to be conducted to determine further progressions in this trend.

Notably, the design used in this manuscript did not consider any architectural modification that might occur in mandibular bones. To verify the clinical application potential of PCL/HA materials, a PCL + 10% HA filament was chosen as an example and produced with material-extrusion-based 3D printing technology for biomimetic bone (Figure 1) as an inexpensive and effective material. A 3D model of a patient-specific implant was designed (Materialise Mimics Innovation Suite and Prusa Slicer software). The model was printed using a gyroid infill pattern with 50% infill density (Prusa Mini printer). The combination of PCL and HA showed promising potential for clinical applications, as seen from the tests mentioned above on PCL/HA scaffolds. In this case, material-extrusion-based 3D printing technology enabled the fabrication of a complex PCL/HA personalized implant. In addition, the relatively low cost of PCL/HA materials will benefit more patients who need bone defect reparations due to tumors and trauma. Additionally, in this case, we successfully demonstrated the feasibility of using a well-described material for thermal filament production and scaffold fabrication, using entry-level equipment translatable to hospital environments in lower-income countries for bone applications.

## 5. Conclusions

PCL composite filaments with different HA ratios were successfully produced using a solvent-free approach to print scaffolds, using a Prusa desktop 3D printer. These scaffolds exhibited constant, interconnected pore sizes, and favorable characteristics, including favorable wettability, compressive strength, and morphology. In conclusion, the reported fabrication parameters are suitable for supporting the further use of filament and scaffold fabrication, which will allow translatability for in-house implants fabrication in hospital settings, utilizing inexpensive machines and standard FDA-approved materials.

## Figures and Tables

**Table 1:** Parameters of the filament fabrication.

Groups	Temperature Gradients/°C				Extruder RPM & Filament (mm/s) Speed
	T4	T3	T2	T1	
PCL	60	64	64	62	2.0 (6.7)
PCL + 5% HA	61	66	66	67	2.9 (10.3)
PCL + 10% HA	69	70	70	69	2.5 (9.7)
PCL + 15% HA	67	67	66	65	2.4 (8.8)
PCL + 20% HA	65	67	67	65	2.5 (9.7)
PCL + 25% HA	61	66	67	66	2.0 (6.7)

RPM: revolution(s) per minute. extruder rotational speed. Filament extrusion speed (mm/s) is expressed in brackets. T1–T4: Four separate zones’ temperature in the filament-maker.

**Table 2:** Parameters used for 3D printing for six groups’ scaffolds.

Groups	Material Extrusion Printing Parameters			
	Nozzle (°C)	Speed (mm/s)	Heated Bed (°C)	Flow Factor (%)
PCL	174	100	30	95
PCL + 5% HA	175	100	30	95
PCL + 10% HA	175	110	30	95
PCL + 15% HA	185	100	30	95
PCL + 20% HA	198	120	30	100
PCL + 25% HA	205	110	30	100

## Data Availability

The data of the study are all included in this study (Supplementary). Metadata are available upon reasonable request to the corresponding author.

## Supplementary Materials

The following supporting information can be downloaded at: https://www.mdpi.com/article/10.3390/polym14040669/s1, Figure S1: Suitable and not suitable filaments. Figure S2: Examples of non-suitable scaffolds. Figure S3: All suitable scaffolds. Table S1: Filament fabrication tested. Table S2: Scaffold printing parameters tested.
