# Supplementary material for: Fabrication and Characterization of PCL/HA Filament as a 3D Printing Material Using Thermal Extrusion Technology for Bone Tissue Engineering
Source: Polymers (Basel). 2022 Feb 11;14(4):669. doi: 10.3390/polym14040669 (PMC8879030; doi:10.3390/polym14040669)
Supplement: Supplementary file 1 [file polymers-14-00669-s001.zip › polymers-1543177-supplementary.pdf]

Supplementary Materials:

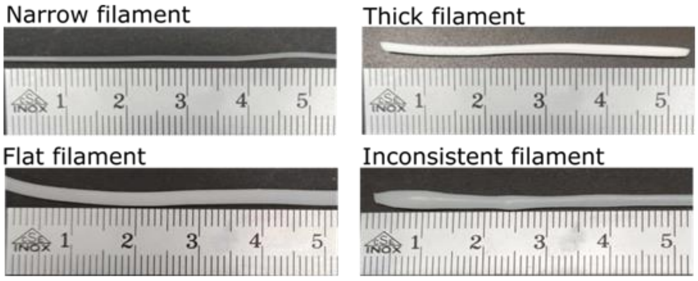

Not suitable filaments

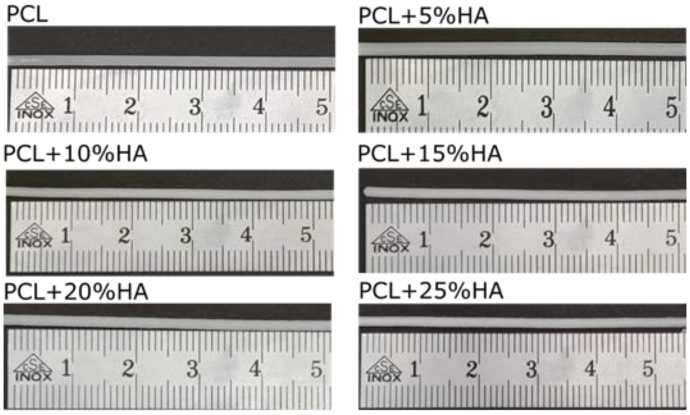

Suitable filaments

Figure S1: Suitable and not suitable filaments.

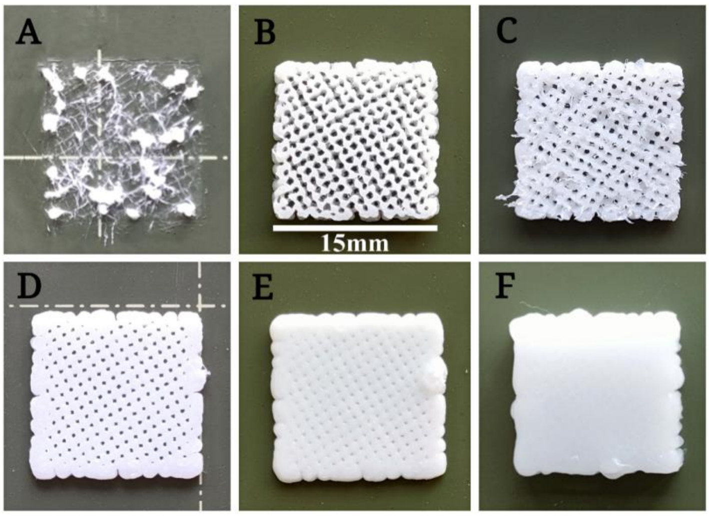

Figure S2: Examples of non-suitable scaffolds. (A) Little extrusion: PCL+25%HA 198–202 °C. (B) Not enough extrusion: filament interruption PCL+15%HA 182–184 °C. (C) Material sticks around the nozzle, causing midway clog: bottom layers are ok but top layers show poor filament extrusion: PCL+20%HA at 110 mm/s speed 196–198 °C. (D) Small pore size (<550 µm): PCL+15%HA 186–187 °C. (E) Small pore size due to extruded material melted close together: PCL+20%HA 199–

200 °C. (F) Extruded material melt together due to too high temperature: PCL 200℃.

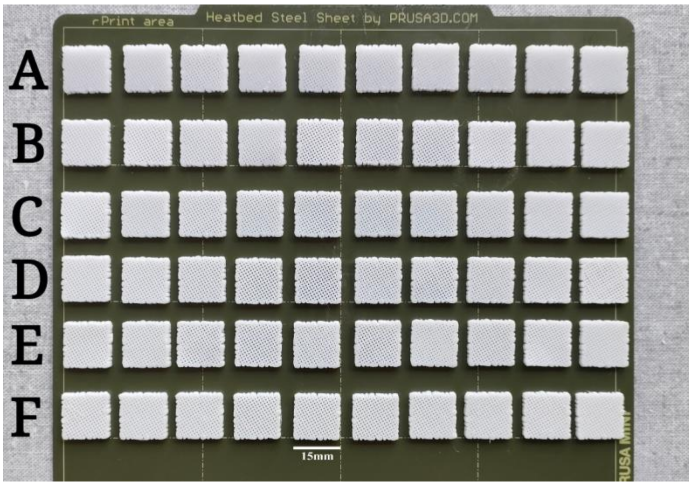

Figure S3: All suitable scaffolds (n = 10). (A) PCL scaffolds. (B) PCL+5%HA. (C) PCL+10%HA. (D) PCL+15%HA. (E) PCL+20%HA. (F) PCL+25%HA.

Table S1: Filament fabrication tested

Groups	T4	T3	T2	T1	Extruder (RPM) & filament (mm/s) speed	Filament results
PCL	66	66	66	66	5 RPM—22.7 mm/s	Thick diameter
	65	65	65	65	4.5 RPM—14.5 mm/s	Thick diameter
	64	64	64	64	→......→2 RPM—6.7 mm/s	Diameter proper but flat
	63	64	64	63	2 RPM—6.7 mm/s	Diameter proper but flat
	62	64	64	62	2 RPM—6.7 mm/s	Diameter proper but slightly flat
	61	64	64	62	2 RPM—6.7 mm/s	Diameter proper but slightly flat
	60	64	64	62	2 RPM—6.7 mm/s	Diameter consistent
+5%HA	60	64	64	62	5 RPM—22.7 mm/s	Extrudes slowly
	61	65	65	63	5 RPM—22.7 mm/s	Extrudes slowly
	62	66	66	65	5 RPM—22.7 mm/s	Thick diameter
	62	66	66	65	→......→3RPM—11.5 mm/s	Inconsistent diameter
	61	66	66	67	2.9 RPM—10.3 mm/s	Consistent diameter
+10%HA	62	66	66	67	5 RPM – 22.7 mm/s	Extruder motor limit reached
	65	69	69	70	5 RPM – 22.7 mm/s	Extruder motor limit reached
	68	69	69	70	5 RPM – 22.7 mm/s	Thick diameter
	69	70	70	71	→.....→2.5 RPM—9.7 mm/s	Diameter proper but flat
	69	70	70	70	2.5 RPM—9.7 mm/s	Diameter proper but flat
	69	70	70	69	2.5 RPM—9.7 mm/s	Consistent diameter
+15%HA	69	70	70	69	5 RPM—22.7 mm/s	Thick diameter
	69	70	70	69	→......→2.5 RPM—9.7 mm/s	Soft and flat filament
	68	69	69	68	2.5 RPM—9.7 mm/s	Soft and flat filament
	67	68	68	67	2.5 RPM—9.7 mm/s	Soft and flat filament
	67	67	67	66	2.5 RPM—9.7 mm/s	Diameter proper but flat
	67	67	67	65	2.4 RPM—8.8 mm/s	Diameter proper but flat
	67	67	66	65	2.4 RPM—8.8 mm/s	Consistent diameter
+20%HA	67	67	66	65	5 RPM—22.7 mm/s	Thick diameter
	67	67	66	65	→......→3RPM—11.5 mm/s	Slightly thick
	67	67	66	65	2.5 RPM—9.7 mm/s	Inconsistent diameter

	67	67	67	66	2.5 RPM–9.7 mm/s	Diameter proper but flat
	67	67	67	65	2.5 RPM–9.7 mm/s	Diameter proper but flat
	66	67	67	65	2.5 RPM–9.7 mm/s	Diameter proper but slightly flat
	65	67	67	65	2.5 RPM–9.7 mm/s	Consistent diameter
+25%HA	65	67	67	65	5 RPM–22.7 mm/s	Thick diameter
	65	67	67	66	→.....→2 RPM–6.7 mm/s	Diameter proper but flat
	65	67	67	66	2 RPM–6.7 mm/s	Diameter proper but flat
	61	66	67	66	2 RPM–6.7 mm/s	Diameter consistent

RPM: Revolution Per Minute

Table S2: Scaffold printing parameters tested

Groups	T/°C	Success	Reason for success or failure
PCL	170–172	N	Little filament extrusion due to low temperature (A)
	173	N	Not enough filament extrusion due to slightly low temperature (B)
	174	Y	Good filament extrusion
	175	N	Small pore size (High temperature leads to high fluidity of extruded material and cannot be cooled in time) (D, E)
+5%HA	174	N	Not enough filament extrudes out due to slightly low temperature (B)
	175	Y	Good filament extrusion
	176	N	Small pore size (D, E)
+10%HA	175	N	Material sticks around the nozzle at 100 speed (C)
	175	Y	Good filament extrusion at 110 speed
	176	N	Small pore size (D, E)
+15%HA	175–181	N	Little filament extrudes out (A)
	182–184	N	Not enough filament extrusion (B)
	185	Y	Good filament extrusion
	186–187	N	Small pore size (D, E)
+20%HA	187–195	N	Little/Not enough filament extrusion at 100 speed (A, B)
	196–198	N	Material sticks around nozzle, causing midway clog at 110 speed (C)
	198	Y	Good filament extrusion at 120 speed
	199–200	N	Small pore size (D, E)
+25%HA	198–202	N	Little filament extrusion at 120 speed (A)
	203–205	N	Not enough filament extrusion at 120 speed (B)
	205	Y	Good filament extrusion at 110 speed
	206	N	Small pore size (D, E)

Y: Yes; N: No. Speed: mm/s. (A, B, C, D, E): Reference to Supplementary Figure S2
